# Fluorescence in Direct Dental Resin-Based Composites and Natural Teeth: A Narrative Review

**DOI:** 10.3390/dj14090535

**Published:** 2026-08-26

**Authors:** Thomas Corfield, Denice Higgins

**Affiliations:** 1 School of Dentistry, Adelaide University, Adelaide, SA 5000, Australia; 2 Forensic Odontology Unit, Adelaide University, Adelaide, SA 5000, Australia; denice.higgins@adelaide.edu.au

**Keywords:** forensic, odontology, fluorescence, composites, resin composite, aesthetics, biomimetics, ultraviolet

## Abstract

**Background/Objectives**: Human teeth naturally fluoresce due primarily to organic components within dentine, contributing to their vitality and appearance under ultraviolet (UV) light. Contemporary resin composites increasingly incorporate fluorescent agents to reproduce this property and improve aesthetic integration with natural dentition. This narrative review examines the fluorescence characteristics of natural teeth and direct resin composites, evaluates methods used to assess dental fluorescence, and identifies factors influencing fluorescence stability and biomimetic performance. **Methods**: Peer-reviewed studies published between 2004 and 2025 investigating fluorescence in human teeth and direct resin-based composites under UV, visible or infrared illumination were reviewed. Studies focused on indirect restorative materials, non-human specimens or caries detection alone were excluded. **Results**: Natural teeth generally exhibit fluorescence emission peaks between 410 and 500 nm, with dentine demonstrating greater fluorescence intensity than enamel. Composite resins commonly incorporate rare-earth oxide fluorophores and often display similar emission wavelengths (approximately 450–485 nm), although fluorescence intensity varies considerably among brands and shades. Material fluorescence is influenced by composition, aging and environmental exposure. Spectrofluorometry provides highly accurate quantitative assessment, whereas photographic and quantitative light-induced fluorescence (QLF) methods offer greater clinical applicability. Despite advances in fluorescence mimicry, variability among contemporary materials generally continues to permit differentiation from natural tooth. **Conclusions**: Although substantial progress has been made in reproducing natural tooth fluorescence, significant variability and temporal instability remain among contemporary composite materials. Fluorescence-based methods therefore remain useful for restoration identification. However, continued improvements in biomimetic performance may reduce their future effectiveness. Greater standardisation of fluorescence assessment and investigation of alternative detection approaches are needed to support clinical and forensic applications.

## 1. Introduction

In recent years, fluorescence has become a growing consideration in resin-based restorative materials, being frequently marketed as a biomimetic property that enhances aesthetic integration under a range of lighting conditions, including natural daylight and ultraviolet-rich environments [1,2,3,4,5,6]. As clinicians are exposed to growing claims regarding the fluorescence performance of restorative materials, understanding the extent to which these materials replicate natural tooth fluorescence is increasingly necessary [1,7]. Despite this commercial and clinical interest, there still remains no clear consensus on acceptable fluorescence characteristics, long-term stability or standardized measurement methodologies [8,9]. In addition, evidence concerning the fluorescence properties of natural teeth, restorative materials and their clinical implications remains fragmented. A contemporary review is therefore warranted.

Fluorescence is an optical phenomenon in which a substance absorbs light at one wavelength and re-emits it at a longer one [10,11]. Human teeth naturally exhibit this by absorbing ultraviolet (UV) light and emitting visible light, which renders them bluish-white under UV illumination. This contributes to natural vitality and luminescence in daylight containing ultraviolet wavelengths [4,11,12,13]. This intrinsic luminescence is primarily attributed to organic components, with dentine typically displaying significantly greater fluorescence intensity than enamel due to higher organic content [4,12,13].

To reproduce the appearance of natural teeth, modern composite resins are formulated to mimic key optical properties, such as translucency and opalescence, fluorescence and hue [10,11,12]. Inadequate fluorescence can result in visible contrast between restorations and teeth under UV-rich lighting conditions, including nightclub environments and photography flashes [4,9,10,11,12]. As a consequence, matching the fluorescence of natural teeth remains an important requirement in achieving aesthetic integration across varying lighting environments [4,9,10,11,12,14].

Material fluorescence is also significant in forensic dentistry [15,16]. When restorations are well matched, differentiating them from natural teeth remains technically challenging [6,17]. Fluorescence-based methods offer a non-invasive, objective solution [5,6,11,17]. For example, UV light sources and quantitative light-induced fluorescence (QLF) technology can enhance detection accuracy [6,17]. Furthermore, advancements in digital photography, such as DSLR cameras with specific band-pass filters, permit reliable documentation and intensity analysis [14,17].

While these techniques can enhance forensic and clinical evaluation, manufacturers are refining composite optical properties by incorporating UV fluorescing pigments to imitate natural teeth [4,9,11,13]. While beneficial to patients, this trend increasingly compromises material detection necessary for clinical documentation and forensic identification [5,6,9,11,17,18]. Although currently mitigated by inconsistencies between materials and teeth [4,5,9,10,11,12,14], existing differentiation techniques may eventually become ineffective [19].

### Aims and Rationale

Given evolving challenges, particularly the potential for improved aesthetic mimicry to reduce restoration visibility under fluorescence methods, the dental fluorescence landscape requires reassessment. The primary objective is determining the ongoing efficacy of UV or other fluorescence-based methods in differentiating composite materials from natural dentition and highlighting alternative techniques. This review aims to:

1. Review the reported fluorescence properties of human enamel, dentine and commercial composite resins.
2. Examine and compare methodologies measuring dental fluorescence in laboratory and clinical settings.
3. Explore factors influencing material fluorescence, such as composition, aging and layering.

A narrative review methodology was selected because the literature is heterogeneous in terms of study designs, fluorescence measurement methods, excitation wavelengths, reporting metrics and outcome measures. As a result, the objective was to provide a broad synthesis of the current evidence rather than conduct a quantitative evaluation or formal evidence appraisal. As with all narrative reviews, the findings may be influenced by selection bias and are dependent on the scope of the literature identified. Therefore, conclusions should be interpreted as an overview of current knowledge, rather than a comprehensive systematic assessment. 

## 2. Materials and Methods

This narrative review examined peer-reviewed studies (2004–2025) assessing human tooth and direct composite fluorescence under UV, visible or infrared light. Only studies using human teeth and direct composite resins were included. Research on indirect materials, non-human teeth or solely caries detection was excluded.

As a narrative review, the purpose of the literature search was to identify representative and influential studies relevant to the review objectives, rather than to perform a comprehensive systematic evidence synthesis. Searches were undertaken in PubMed/MEDLINE, Scopus and Web of Science Core Collection for articles published between January 2004 and March 2025. Search terms included combinations of keywords relating to dental fluorescence, tooth fluorescence, resin composites, restorative materials, ultraviolet light, fluorescence imaging, spectrofluorometry and quantitative light-induced fluorescence.

Peer-reviewed studies evaluating fluorescence in human dental tissues or direct resin-based composite materials were considered for inclusion. Literature searches were conducted in PubMed, Scopus and Google Scholar between January and March 2025. Searches focused on fluorescence properties of natural teeth and direct resin composites. Articles published in English between 2004 and 2025 were considered. Search terms included combinations of “dental fluorescence”, “tooth fluorescence”, resin composite fluorescence”, “fluorescence restorative materials”, “ultraviolet fluorescence dentistry”, “quantitative light-induced fluorescence”, “forensic fluorescence density” and “fluorescent dental composite”. Grey literature was not systematically searched. Reference lists of included articles were reviewed to identify additional relevant publications. Studies focused exclusively on indirect restorative materials, non-human specimens, caries detection, conference abstracts and non-peer-reviewed sources were excluded. Relevant findings relating to fluorescence characteristics, assessment methodologies, and factors affecting fluorescence behaviour were synthesised narratively owing to substantial methodological heterogeneity among studies. As this review was intended as a narrative synthesis rather than a systematic evidence appraisal, a formal risk-of-bias assessment was not performed.

## 3. Results

Seventeen studies meeting the review objectives were identified and are summarised in Table 1, Table 2, Table 3 and Table 4.

### 3.1. Excitation Sources and Methodologies

(Table 1) Most studies employed UV light as the primary excitation source, with spectrometric excitation wavelengths ranging from 375 nm to 410 nm [6,13,20,21]. While spectrometric analysis often focuses on narrow bands, some imaging used a wider excitation range (400–500 nm), spanning from UV-A to visible violet/blue light [6,18]. This broader range (400, 430, 450, 470 nm) was preferred to enhance relative fluorescence differences between teeth and materials for visual discrimination, despite potentially lower intensity [18]. Notably, 405 nm, frequently delivered by safer LED sources, emerged as a key visible-light excitation wavelength [17]. The most-cited excitation wavelength was approximately 398 nm [11,12], reliably producing peak emissions around 485 nm. Other wavelengths included 375, 395, 405 and 430–470 nm [13,14,17,18,21]. Practical tools like filtered flash photography (365–405 nm) were noted for safety and utility [14]. Infrared excitation or emissions were not evaluated in any reviewed studies.

### 3.2. Methods for Assessing Dental Fluorescence

Most studies used standardised preparation and controlled excitation via spectrometry or calibrated photography, though subjective scoring was also employed [6,12,13,20,21]. Spectrofluorometry, the most common method, provided quantitative intensity and spectral profiles [11,12,13]. Devices like the Ocean Optics USB 4000 and PerkinElmer LS 55 generated relative fluorescence units known as RFU [5,12,13]. With these devices, emission peaks for composites and teeth were typically between 450–485 nm, though intensity and shape did vary [11,12,13,22].

Photography under ultraviolet or violet illumination commonly uses DSLR cameras fitted with band-pass filters (typically 365 or 405 nm) to quantify fluorescence using greyscale, RGB, CIELab and contrast-ratio measurements [4,9,10,14,17]. Compared with relative fluorescence unit (RFU) measurements obtained from spectrofluorometry, photographic techniques offer greater clinical practicality and visual relevance. Quantitative light-induced fluorescence (QLF) combines imaging and objective measurement by converting fluorescence images into numerical greyscale values for analysis [6]. In contrast, some studies employed subjective scoring systems that compared material fluorescence with that of natural enamel, aiming to reflect how fluorescence differences are perceived clinically [20,21].

Reporting both spectral shape and intensity provides better insight. Da Silva et al. (2014) showed composite absorption from 250–450 nm and emissions around 485 nm [12]. Tani et al. (2003) found teeth exhibited broader fluorescence than resins beyond 430 nm [18]. Meller and Klein (2012; 2015) reported composite peaks at 452 ± 9 nm versus broader tooth spectra [5,11]. Studies reporting only intensity values (AU, RFU, RGB) lack spectral detail [6,9,10,14,20]. Spectrofluorometry is precise but clinically impractical [6,11,12]. While photography is accessible, it faces standardization issues [14,17], making correlation with spectrofluorometry difficult [4]. Subjective scoring lacks quantitativeness [6,20]. Nevertheless, all methods demonstrate considerable intensity variation, complicating comparison and aesthetic matching [4,6,11,23].

**Table 1:** Summary of studies’ fluorescence measurements and light sources.

Methodology	Primary Instrument	Excitation Wavelengths Used	Key Characteristics/Limitations
Spectrofluorometry [11,12,13,18,21]	Laboratory Spectrometers (e.g., Ocean Optics, PerkinElmer)	UV: 375 nm, 395 nm, 398 nm Violet/Blue: 405 nm, 410 nm, 430–470 nm	Pros: High precision; quantitative emission spectra; high reproducibility. Cons: Clinically impractical; complex equipment.
Digital Photography [6,9,10,17,18,20]	DSLR Cameras + Band-pass filters	UV-A: 365 nm Violet: 405 nm	Pros: Clinically relevant visualisation; captures ‘real-world’ aesthetic perception. Cons: Less precise; standardisation is difficult; hard to correlate with spectral data.
QLF Technology [6]	Quantitative Light-induced Fluorescence (QLF-D)	Blue: 405 nm	Pros: Balances objective measurement (greyscale values) with visual relevance.
Visual Scoring [20,21]	Human Evaluators	UV/Visible: Various	Pros: Reflects aesthetic perception. Cons: Subjective; lacks quantitativeness.

### 3.3. Natural Tooth Fluorescence Characteristics

(Table 2) Natural teeth generally peak in the 410–500 nm range, often specifically around 440–450 nm [11,12,22]. This sums dentine and enamel spectra. When irradiated with 365 nm light, dentine consistently shows blue fluorescence peaking at 440 ± 10 nm [4,11,12,23,24]. Other sources report dentine peaks between 420 and 450 nm [13,23] or specifically at 406 ± 20 nm when excited at 354 ± 14 nm [11]. Dentine autofluorescence may be influenced by age, with increased collagen and organic matrix enhancing intensity [4,11,23,24].

**Table 2:** Summary of baseline characteristics of natural teeth and fluorescence evaluation techniques.

Feature	Natural Dentine	Natural Enamel	Measurement Methodology (Spectrofluorometry)	Measurement Methodology (Photography/QLF)
Emission Peak	440 ± 10 nm (blue) [22]	440–450 nm (broad band) [22].	Quantitative: Measures relative fluorescence units (RFU/AU) and provides precise spectral profiles [11,12].	Visual: Captures aesthetic perception and provides less precise greyscale/RGB values [10,20].
Intensity	High (stronger than enamel) [10,11,21,22,23]	Low (weaker than dentine) [5,11,12,13,21].	Excitation Sources: Typically uses UV-A (375–398 nm) or Violet/Blue (405–470 nm) [6,10,12,13].	Excitation Sources: Typically uses 365 nm (UV-A) or 405 nm (Violet LED) [6,11,17,21,22].
Primary Cause	Organic components (collagen, tryptophan) [6,12,13,21,23,24]	Inorganic/Organic Blend (apatite, lower organic content) [6,12,20,21,23].	Pros: High precision and reproducibility; essential for spectral comparison [5,11].	Pros: Clinically relevant; useful for documenting contrast and aesthetic match [6,17,18,20].
Stability	Stable (may increase with age) [23,24].	Stable [10,23].	Cons: Clinically impractical; complex laboratory setup [6,12].	Cons: Lower precision; difficult to standardise or correlate with spectral data [6,11,12].

Human enamel exhibits lower fluorescence intensity than dentine [4,12,13,14]. Enamel typically displays luminescence peaks in the 350–360 nm, 405–410 nm, and 440–450 nm regions [17,22]. Its spectrum generally presents as a wide band maximising around 450 nm and diminishing toward longer wavelengths [22]. Other reported emission peaks include 450–470 nm, and experimentally, approximately 460, 470 and 480 nm when excited at 375, 395 and 410 nm, respectively [13]. Reported fluorescence depends on the excitation light. For example, teeth absorb light between 250–300 nm to exhibit maximum emissions at approximately 485 nm, with some samples peaking around 490 nm [12]. At longer excitation wavelengths (375, 395, 410 nm), lower emission peaks were recorded [13]. One study reported lower emission peaks between 350 and 450 nm using narrow or broadband UV, but did not specify the wavelength [17]. Regardless, tooth fluorescence is primarily attributed to organic components [4,6,12], with dentine’s greater intensity attributed to higher collagen content and photosensitive amino acids like tryptophan and hydroxypyridine [6,12,23].

### 3.4. Dental Composites Fluorescence Properties

(Table 3) Composite components lack inherent fluorescence; luminescent elements like rare earth oxides (europium, cerium, ytterbium, terbium and thulium) are added [4,6,9,11,21,23,24]. Although exact formulations are proprietary, evidence consistently identifies rare-earth oxides as key additives [4,6,9,12,21,23,24].

**Table 3:** Fluorescence properties of dental composites by brand.

Composite Brand (Manufacturer)	General Observation/Rank	Emission Peak Cited	Key Finding/Rationale for Difference
Durafill-VS	Highest Intensity	~485 nm	Up to 13,539 AU cited; significantly brighter than natural tooth or other composites [12].
Filtek Z350/Z350XT (3M ESPE)	Low Intensity	~485 nm	Closest intensity match to enamel in some studies (1146 AU vs. 1380 AU for enamel) [12].
Opallis	Variable Intensity	450–485 nm	Intensity decreased after 90 days of natural aging [13].
Empress Direct	Variable Intensity	450–485 nm	Intensity decreased after 90 days of natural aging [13].
Charisma	High Fluorescence	Not specified	Ranked as a highly fluorescent material in visual/QLF studies [12].
DenFil	Minimal Fluorescence	Not specified	Ranked as one of the least fluorescent materials [12].
Amelogen Plus	Variable Match	Not specified	Matched natural fluorescence well in some studies, but results were inconsistent across the literature [12].
Filtek Universal	Less Fluorescent	Not specified	Significantly less fluorescent than HRI, Harmonise, or Herculite XRV [12].

Freshly cured composite emissions generally peak similarly to natural teeth, often around 450–485 nm [11,12,14,22,23]. Specifically, studies report maximum emissions at approximately 485 nm when excited at 398 nm [12], and 452 ± 9 nm when excited at 398 ± 5 nm [11]. Others note peaks around 440–450 nm under 365 or 380 nm excitation [14]. However, considerable intensity variation exists [6,11,12,14,23]. Most composite shades display greater maximum fluorescence than dentine or enamel [5,11], with some being up to six times brighter under UV light [21]. Conversely, some composites lack fluorescence and appear dark [9,17,21].

Significant intensity variation exists among brands and shades, highlighting inconsistent fluorophore incorporation [4,6,11,12,14,21,23]. For example, Durafill-VS showed the highest intensity (13,539 AU), while Filtek Z350 showed the lowest (1146 AU), closest to enamel (1380 AU) [12]. Other studies ranked Gradia and Charisma as highly fluorescent, and DenFil as minimal [6]. Amelogen matched natural fluorescence in some studies [10], while Filtek Universal was significantly less fluorescent than HRI, Harmonise, or Herculite XRV [4]. Fluorescence also varies between shades. While some research suggests negligible differences at wavelengths less than 500 nm [18], other studies demonstrate that special-modification shades (transparent, amber, intensive-white) fluoresce more than dentine or enamel shades [5,12]. Even among A1–A3 shades, significant visual differences remain under UV light [4].

### 3.5. Composite Brands and Fluorescence Characteristics

Filtek Z350 and Z350XT (3M ESPE) were studied most frequently [6,12,13], followed by Opallis and Empress Direct [13]. Additional composites included DenFil, Premisa, Grandio, Charisma, Gradia Direct Posterior [6], Esthet-X, Amelogen Plus and Durafill-VS [12]. One survey assessed 46 resin-based composites [21]. Studies evaluated enamel, dentine and various shades and viscosities [12,13,20,21]. Findings demonstrate that while composites generally mimic natural tooth wavelengths, intensities vary widely and often exceed natural tooth levels [6,11,12,13,21]. These differences influence visual matching and detectability [6,12,21]. Moreover, intensity changes over time; some materials increase in fluorescence while others decrease [13]. While most composites remain visually distinguishable under UV/violet light, not all are consistently detectable [17,20,21].

### 3.6. Factors Affecting Composite Fluorescence

(Table 4) To replicate complex organic tooth fluorescence, manufacturers incorporate artificial fluorophores into the glass filler phase [1,25]. Variations likely stem from filler content, filler-to-resin ratios and matrix composition [10,21,23]. While some parameters like filler size and shape may not significantly affect fluorescence [4,11], others argue that both inorganic filler and organic matrix compositions contribute [20]. Matrix viscosity may also affect stability [26]. Studies using fluorescing dyes showed that in highly viscous adhesives, limited dye mobility maintains intensity, whereas low-viscosity solvents, such as ethanol, increase interactions leading to fluorescence loss [21,26]. Although dyes differ from commercial fluorophores, this suggests adhesive properties may play a role. Furthermore, manufacturing changes influence fluorescence [2,27], with evidence that different batches of Admira Fusion X-tra demonstrate spectral differences that suggest formulation drift [21].

**Table 4:** Factors influencing composite fluorescence.

Factor	Influence on Fluorescence	Mechanism/Observation
Material composition	Primary Driver	Rare-earth oxides in the glass filler and organic matrix composition dictate fluorescence. Proprietary formulations prevent thorough understanding [5,6,9,11,13,21,23,24].
Filler Content/Ratio	Contributes to Variability	Variations in filler-to-resin ratio and fluorophore concentration affect intensity and spectral properties [5,6,10,11,20,21,23,24].
Aging and degradation	Decreases intensity	Fluorescence generally degrades over time due to polymer degradation and environmental exposure [10,13,23,24]. Exception: Z350XT showed increased intensity in one study [12].
Hydration	Decreases intensity	Water absorption leads to scattering or quenching mechanisms, diminishing fluorescence [13,17].
Manufacturing batches	Inconsistency	Different batches of the same product (e.g., Admira Fusion) showed different spectral properties, indicating formulation drift [5,9,21].
Shade selection	Variable effect	Special shades (transparent, amber, white) often fluoresce much more intensely than standard dentine/enamel shades [5,12,17].

### 3.7. Temporal Stability and Challenges in Fluorescence Mimicry

Mimicking stable natural tooth fluorescence, which may increase with age due to collagen deposition, is difficult [12,23]. Many composites exhibit reduced fluorescence with age [4,10,11,13,17,23]. However, exceptions exist. Z350XT, for example, showed increased intensity after 90 days, while Opallis and Empress Direct decreased [13]. In contrast, simulated aging through UV irradiation, thermal exposure and water storage generally reduces fluorescence intensity [4,13]. In both cases, changes are often pronounced within the first 30 days, most probably as a result of polymer degradation [13,23] and hydration-induced scattering or quenching [17].

## 4. Discussion

This review examined tooth and composite fluorescence, measurement methodologies and influencing factors, with findings suggesting that while advances in mimicking natural tooth fluorescence are significant, challenges remain. Indeed, although material fluorescence is frequently promoted as an aesthetic property, evidence suggests substantial variation among commercially available materials. Consequently, clinicians cannot assume that materials labelled as fluorescent will demonstrate comparable fluorescence behaviour or long-term stability.

The current evidence demonstrates a growing understanding of fluorescence behaviour in natural teeth and composite resins. However, this knowledge remains fragmentary because of inconsistent methodologies, differing excitation protocols, and variable means of measurement. While the optical characteristics of commonly used fluorescence have been described, comparatively little is known regarding long-term fluorescence stability, layered restorations, emerging universal composites and alternative excitation technologies.

The increasing commercial emphasis on fluorescence also raises questions regarding standardisation. At present, there is no universal benchmark that defines acceptable fluorescence relative to natural teeth, which further complicates comparisons between products.

Many modern composites exhibit fluorescence intensities and emission peaks comparable to enamel and dentine (450–485 nm) when excited by UV-A or violet light [11,12,14]. While reflecting progress in mimicry, intensity varies widely between brands, shades and batches [4,6,21]. Furthermore, temporal performance degrades with aging and environmental exposure [13], suggesting stability is yet to be controlled.

A diverse range of techniques was used. Spectrofluorometry provides precise quantitative data [11,13] but lacks portability. Photographic techniques are more clinically relevant [14,17] but less precise. Few studies captured spectral shape and intensity. Relying on greyscale or colour values may inaccurately suggest a match if materials share brightness but differ in spectral shape [4,6]. Ultimately, lack of standardisation makes comparing studies difficult.

The development of standardised reporting guidelines for dental fluorescence studies would substantially improve study comparability. Key elements should include reporting of excitation wavelength, emission wavelength range, specimen preparation, aging conditions, calibration procedures, fluorescence units and imaging parameters. Such standardisation would facilitate meta-research and improve interpretation of future investigations.

Improved mimicry poses challenges for material detectability. Several studies demonstrate that better mimicry compromises detection under conventional UV examination [4,6,18]. Clinically, non-detection affects retreatment planning; forensically, it compromises identification. However, currently reported variability remains a temporary advantage, as materials fluorescing too brightly or dimly are distinguishable [9,21]. This inconsistency cannot be relied upon indefinitely.

Factors affecting composite fluorescence include filler content, monomers, fluorophore choice, and layering [5,20,23]. Exact formulations remain proprietary [4,11]. Discrepancies between batches point to formulation drift [21]. Furthermore, aging, hydration and staining agents influence stability, often leading to quenching [17,23].

Gaps in the literature include a lack of standardisation in measurement protocols and reference data [21]. No reviewed studies employed infrared excitation despite its increasing use in bioimaging. Fluorescence behaviour under layered application is underexplored. There is also a lack of manufacturer transparency that impedes investigation in the first instance. Without accurate compositional data, correlating fluorescence with specific formulations is particularly difficult. Future research should prioritise standardised protocols, explore alternative excitations like near-infrared, and advocate for transparency to improve reproducibility. Real-time diagnostic imaging combining spectrometric precision with visualisation would likely improve differentiation.

This review had several limitations that need to be acknowledged. Although this review was conducted and reported in accordance with the Scale for the Assessment of Narrative Review Articles (SANRA) framework, it remains a narrative review rather than a systematic one. Consequently, the search strategy was designed to identify representative and relevant literature rather than all potentially eligible studies, and a formal risk-of-bias assessment was not undertaken. In addition, substantial heterogeneity existed among the included studies with respect to excitation wavelengths, fluorescence measurement techniques, reporting metrics, specimen preparation protocols, and aging methodologies, which hinders direct finding comparisons. The review was also restricted to English-language peer-reviewed publications and did not systematically evaluate grey literature, which may have resulted in omission of relevant information. The findings, therefore, should be interpreted as a qualitative synthesis of the current literature rather than a definitive quantitative assessment of fluorescence characteristics in natural teeth and contemporary composite resins. The SANRA checklist used to guide reporting is provided in Supplementary File S1.

## 5. Conclusions

Dental fluorescence studies are complicated by unstandardized protocols, proprietary formulations and natural tooth variability. While manufacturers aim for optical similarity, consistently achieving it remains complex. Significant strides have been made, with some composite shades achieving emission values comparable to natural structures. However, inter-brand variability and aging effects persist.

Generally, reported variability currently permits restoration differentiation under UV illumination. Current fluorescence-based identification remains effective largely because composite fluorescence remains inconsistent between brands and shades. While some composites mimic enamel closely, others show distinct intensity differences, meaning even those with similar spectral colour may appear darker or brighter than teeth. Ultimately, combined approaches like QLF and photography may be necessary to assess indistinguishable composites. While UV fluorescence remains useful, material improvements challenge its reliability, necessitating innovative detection methods for clinical and forensic differentiation.

## Data Availability

No new data were created or analyzed in this study. Data sharing is not applicable to this article.

## Supplementary Materials

The following supporting information can be downloaded at: https://www.mdpi.com/article/10.3390/dj14090535/s1, Supplementary File S1: SANRA Checklist (Scale for the Assessment of Narrative Review Articles).
