# Supplementary material for: Fluorescence in Direct Dental Resin-Based Composites and Natural Teeth: A Narrative Review
Source: Dent J (Basel). 2026 Aug 26;14(9):535. doi: 10.3390/dj14090535 (PMC13605867; doi:10.3390/dj14090535)
Supplement: Supplementary file 1 [file dentistry-14-00535-s001.zip › dentistry-4466867-supplementary.pdf]

Supplementary File S1

SANRA Checklist (Scale for the Assessment of Narrative Review Articles)

Manuscript Title: Fluorescence in Dental Composites and Natural Teeth. A Narrative Review of Optical Characteristics and Material Differentiation

SANRA Item	Description	Compliance Statement
**1. Justification of the Review's importance for readership**	Rationale“The rationale and relevance of the review topic should be clearly explained.”	Dental fluorescence is increasingly relevant in restorative dentistry, aesthetic dentistry, clinical diagnostics and forensic odontology. As composite materials are increasingly engineered to mimic the optical behavior of natural teeth, understanding the current capabilities and limitations of fluorescence-based differentiation methods has important implications for both clinical practice and forensic identification. The review was undertaken to synthesise current knowledge and identify emerging challenges in this rapidly evolving field.
**2. Statement of concrete aims or formulation of questions**	“The review should clearly identify its objectives.”	The manuscript includes an "Aims and Rationale" section outlining the primary objective of assessing the efficacy of fluorescence-based methods for differentiating composite materials from natural dentition. Specific review aims are listed as bullet points.
**3. Description of the literature search**	“Sources and search approach should be described.”	The methodology section describes the databases searched, eligibility criteria, search period (2004-2025), inclusion and exclusion criteria, search terms and any supplementary bibliography searching performed.
**4. Referencing**	“Statements should be supported by relevant and	The manuscript references primary studies investigating fluorescence characteristics of natural teeth, restorative materials, assessment methodologies, aging effects and forensic applications. Citations are

	appropriate references.”	provided throughout the text and supporting tables.
**5. Scientific reasoning**	“Evidence should be critically analysed and interpreted.”	Findings are synthesised across multiple studies, with discussion of methodological differences, variability in fluorescence measurements, aging effects, clinical implications, forensic relevance and limitations of current approaches. Contradictory findings and research gaps are identified where relevant.
**6. Appropriate presentation of data**	“Results should be organised and presented clearly.”	Key findings are summarised narratively and through tabulated presentation of excitation sources, fluorescence characteristics, influencing factors and assessment methodologies. Discussion integrates findings into clinically and scientifically relevant themes.

Summary

The manuscript was prepared in accordance with the principles of high-quality narrative review reporting and addresses all six SANRA domains through explicit reporting of review objectives, literature selection methodology, evidence synthesis, critical discussion and identification of future research priorities.
